# Structure Guides Function: Beyond Pairwise Neural Interactions

**DOI:** 10.1002/cne.70213

**Published:** 2026-09-30

**Authors:** Alexander Maier

**Affiliations:** ^1^ Department of Psychology Vanderbilt University Nashville Tennessee USA

**Keywords:** higher arity, higher order, hypergraphs, neuronal information processing

## Abstract

A central theme in Jon Kaas’ research, celebrated in this issue, is the relationship between brain structure and function. As a tribute, we examine how certain neuroanatomical structures enable higher order (higher arity) functions whose joint effects cannot be recovered from pairwise measurements. Specifically, we argue that neurons exploit coincident input detection to realize higher order operations that cannot be derived by studying any of the pairwise combinations in isolation. Pairwise statistics can miss these irreducible higher order (more‐than‐pairwise) effects. We advocate for an extension of graph‐based models to hypergraphs to formally capture and analyze these irreducible higher order neural interactions. Based on several recent studies, we discuss both the applicability and benefits of higher order neural data analyses.

## Graphical abstract

Some neurons fire more to synchronous inputs than combination of isolated inputs predicts. This synergistic effect is largely invisible to pairwise measurements. We thus propose to represent neural circuits as hypergraphs, where edges connect three or more interacting neuronal structures, in addition to using lines for connected pairs.

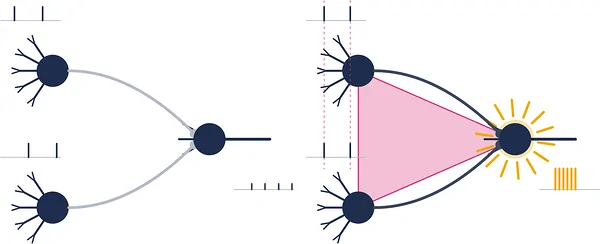

## 1 Introduction

This special issue honors the enduring scientific legacy of Jon Kaas. One of Jon's many impactful contributions to systems neuroscience is the discovery of a close relationship between neuroanatomical connectivity and functional cortical organization (Allman and Kaas 1971, 1975; Kaas 1983; Pons et al. 1991; Kaas and Hackett 2000; Jain et al. 2025). In what follows, we argue that Jon's insights were not only foundational to the development of modern systems neuroscience (e.g. Snyder and Bauer 2019) but continue to inspire novel discoveries. In particular, we discuss how convergent neuronal structures can give rise to a form of irreducible interactions (Zapata‐Carratala and Arsiwalla 2022; Robiglio et al. 2025)—those whose joint effect cannot be recovered from measurements of the constituent pairs. Note that this notion of irreducibility is distinct from related notions, such as decomposition into pairwise components, which at least for functions is always possible (Sierpiński 1945).

## 2 Irreducible Higher Order (Higher Arity) Relations

Neuronal causal interactions go beyond two neurons forming a pairwise relation, such as an upstream neuron causing activation in a downstream neuron (Grasso, Albantakis, et al., 2021). For example, three or more neurons can give rise to a combined effect, which is often referred to as “higher order” (Santoro et al. 2024; Battiston et al. 2020, 2021, 2025; Bianconi 2021; Torres et al. 2021; Bick et al. 2023; Kurkin et al. 2026; Boccaletti et al. 2023; Niedostatek et al. 2025; Giusti et al. 2016; Millán et al. 2025; Tabar et al. 2024; Chung et al. 2025). A related mathematical term is “higher arity” (Figure 1). Arity describes the number of input arguments to a function, or the number of elements that stand in relation to each other, with *binary* denoting a pairwise relation, *ternary* denoting a three‐way relation, and so forth (Zapata‐Carratala and Arsiwalla 2022).

**FIGURE 1:**
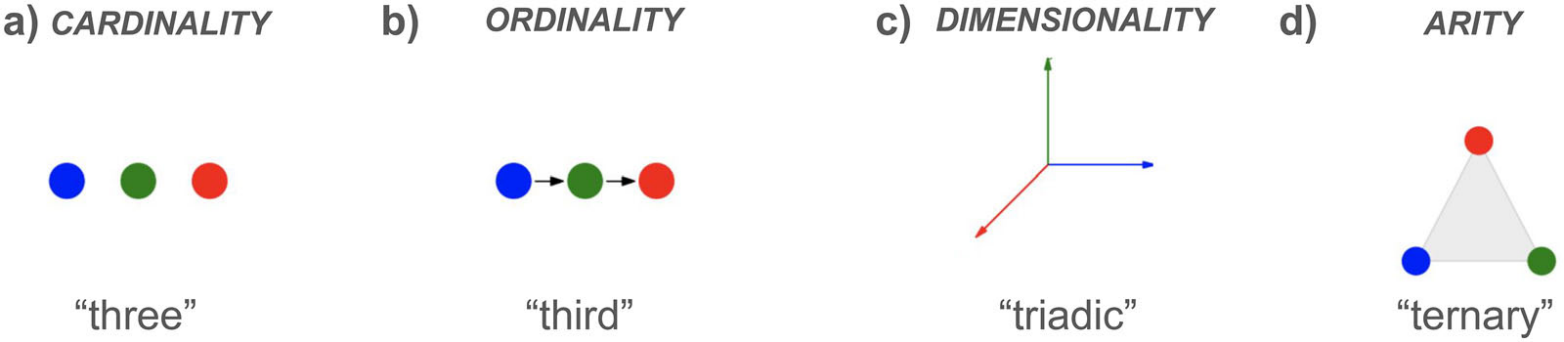
Four distinct mathematical notions of “three‐ness.” (a) Cardinality describes quantity, such as elements of a set. (b) Ordinality describes rank or position within a sequence. (c) Dimensionality describes the number of independent parameters used for representing a structure. (d) Arity is the number of operands or arguments that serve as input to a function, operation, or relation. Pairwise relations are binary. Arity higher than two (n‐arity with n > 2) is in this context considered “higher order” in the sense that it involves *more‐than‐pairwise* relations.

Many mathematical operations, such as the arithmetic operations of addition, subtraction, multiplication, and division, are pairwise (*binary*) in that they only allow for a pair of numbers to be operated on (Odrzywołek 2026). Adding or multiplying more than two numbers involves breaking down the operation into a sequence of pairs by using the associative law (e.g., 3 × 5 × 2 = (3 × 5) × 2 = 15 × 2 = 30). Following the above convention, a three‐way relation is called *ternary*, a four‐way relation *quaternary*, and so on (with n‐ary representing the general rule).

Most importantly, both this mathematical definition and the physical reality of these more‐than‐pairwise (ternary and beyond) relations, processes, or operations are that some of them are irreducible, as defined above (Figure 2). In other words, an irreducibly ternary interaction between three neurons cannot be derived by studying any of the pairwise combinations in isolation. In mathematics, the Borromean rings exemplifies this property since each pair of elements does not produce a knot; only simultaneous combination of all three elements prevents the elements from separation (Figure 2a). In physics and engineering, the combustion triangle exemplifies non‐reducibility to pairwise interactions: combining fuel and oxygen (without heat), fuel and heat (without oxygen), or heat and oxygen (without fuel) all fail to ignite. Only when all three of these factors are combined at the same moment and location, does combustion occur (Figure 2b).

**FIGURE 2:**
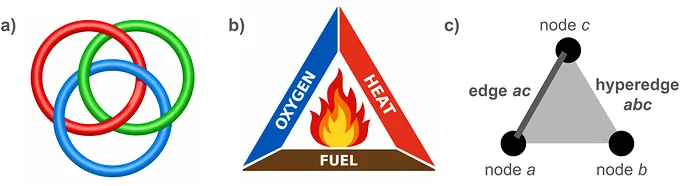
Irreducible higher order (ternary) relations. (a) Borromean rings. When any one of the three loops is cut or removed, the knot falls apart into unlinked loops. (b) Combustion triangle. Fuel, oxygen, and heat must all be present simultaneously. No pairwise subset of these three components is sufficient to predict ignition, only their joint occurrence produces the effect. (c) A hypergraph representing a ternary interaction between three nodes (*a*, *b*, and *c*). Graph nodes (vertices) are depicted as black disks. Standard graph theory consists of pairwise connections (edges) that are represented as lines along the shortest Euclidean distance between two nodes. The black line connecting the leftmost two nodes is an example of such a graph (edge *ac*). Hyperedges connect three or more nodes. The gray triangle that connects all three nodes represents such a non‐pairwise hyperedge (hyperedge *abc*). Hypergraphs are graphs that contain hyperedges. None of the (a)–(c) relations can be reduced to pairwise relations in a way that allows for reconstruction (prediction) of the ternary relation from studying the pairwise interactions in isolation.

The combustion triangle in particular demonstrates that (i) irreducible ternary effects occur in nature, and (ii) studying such irreducible ternary effects from a strictly pairwise perspective is limited. That is, an exhaustive study of the causal consequences of all pairwise combinations of fuel, heat, and oxygen (none of which result in a combustion) would not lead to the prediction that combining all three of these factors results in a combustion. Analogously, studying the brain from a strictly pairwise perspective (i.e., using pairwise statistics) exclusively, risks that certain (i.e., higher order) brain processes fall into a methodological blind spot. Indeed, several studies that tested explicitly whether pairwise statistics alone are sufficient to describe the dynamics of larger neuronal populations concluded that higher order (higher arity) interactions both occur and need to be accounted for (Ganmor et al. 2011; Roudi et al. 2009; Ohiorhenuan et al. 2010).

Mathematically, such irreducible ternary (and beyond) relations can be represented using a hypergraph (Figure 2c). Hypergraphs consist of a limited set of nodes and edges in the same way as regular graphs. The only difference to standard graph theory is that while in graphs edges connect *pairs* of nodes, hypergraphs allow for edges to connect *three or more* nodes. Graphs connect nodes via lines or arrows. Hypergraphs extend this notation by increasing arity. For ternary relations, a ternary “hyperedge” can be visualized as a triangle (surface). A quaternary relation can be represented by a pyramid (volume), and so on. Alternative visual representations include denoting a hyperedge by an outline that encompasses all connected nodes, akin to a rubber band. In either case, the result formally captures higher arity (e.g., ternary or higher relations) and is called a hypergraph.

## 3 Higher Order Neuronal Interactions

How is all of this relevant to systems neuroscience? Donald Hebb is credited with being one of the first theoreticians to consider the potentially synergistic effects of converging neuronal inputs onto the same neuron (Figure 3a). Synaptic strength is also thought of as “synaptic weight,” and probably best formalized as the probability of a postsynaptic neuron firing depending on one or both or the presynaptic neurons being active.

**FIGURE 3:**
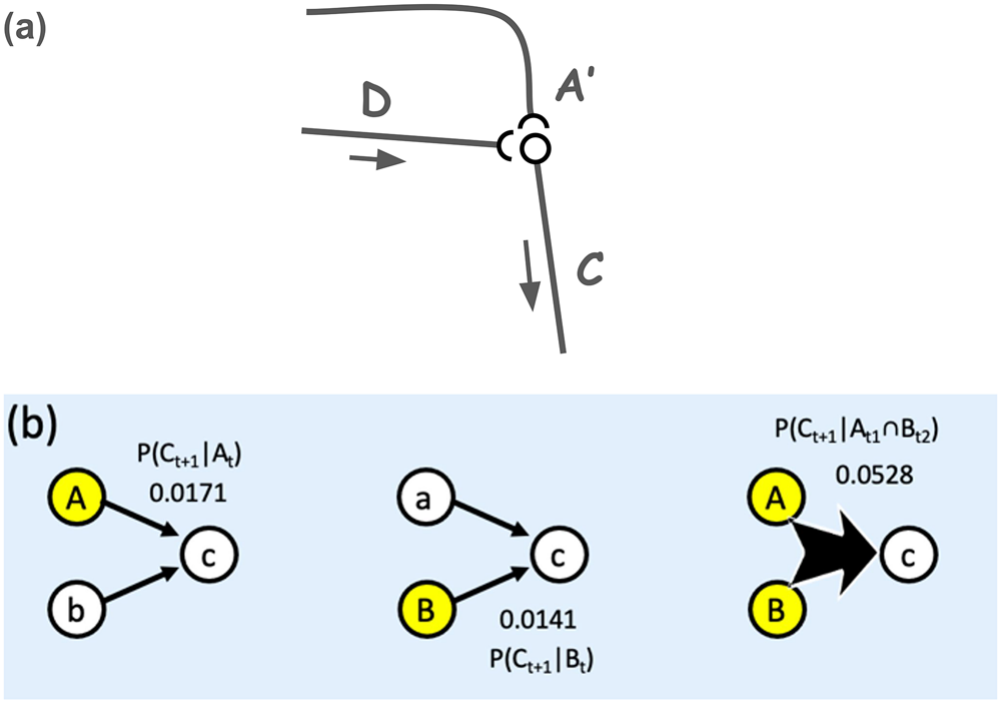
(Ternary) Neuronal information processing. (a) Schematic representation of the original diagram by Donald Hebb illustrating his thoughts on synaptic processing of coincident inputs (modified from: (Seung 2000). (b) Schematic summarizing empirical evidence that coincident inputs nonlinearly increase the likelihood of a postsynaptic neuron firing (adapted from Alonso et al. (1996)). The isolated monosynaptic influences of neurons *a* and *b* were quantified as the conditional probability that neuron *c* fires 8–12 ms after a presynaptic spike. Using conventional graph theory, these pairwise interactions (*a* → *c* and *b* → *c*) are typically represented by directed edges (arrows), where edge thickness encodes synaptic strength. However, when both neurons *a* and *b* were active simultaneously, the probability of neuron *c* firing increased beyond what would be expected from summing or multiplying their individual contributions, indicating a *supralinear* effect. The three neurons thus form a *higher order*, ternary interaction (large arrow: *ab* → *c*) that cannot be fully predicted, described, or explained from its constituent pairwise interactions measured in isolation.

In a landmark study, Alonso and coworkers (1996) demonstrated that following this logic *irreducible ternary* interactions are demonstrable in the mammalian primary visual pathway (Figure 3b). Specifically, they isolated neurons in the primary visual cortex (V1) that received inputs from pairs of lateral geniculate nucleus (LGN) neurons. Activity of all three of these neurons was measured (recorded) extracellularly at the same time. This setup allows for the calculation of synaptic efficacy of each pair of upstream–downstream neurons: whenever one of the two LGN upstream neurons was active, there was a small but reliable increase in the probability of the downstream V1 neuron to become active (following the expected brief synaptic delay). Interestingly, when both LGN neurons were active *at the same time*, the resulting increase in the recipient V1 neuron firing increased *nonlinearly* (i.e., the likelihood of the V1 neuron firing was more than the sum of the pairwise synaptic efficacies/weights).

Subsequent work revealed molecular coincidence detectors at dendrites (Schiller et al. 2000). In this light, the nonpairwise effect observed by Alonso and coworkers is not just a nonlinearity that is “added” after pairwise summation. Instead, it marks a *single physical event* that only exists when two or more inputs arrive within a narrow time window. This process constitutes a genuinely ternary (or higher order) event in the sense of Figure 2: single inputs or two inputs arriving in sequence fail to ignite the higher order process; only two *simultaneous* inputs evoke this unique process.

Thus, just like in the case of the combustion triangle, examining the three‐way relation of neurons by first breaking it into pairs (thus allowing for standard pairwise mathematical operations, such as Pearson correlations or conventional Granger causality) fails to predict the *nonpairwise* synergistic effect that occurs via temporal synchronicity (James et al. 2016; Stramaglia et al. 2014; Williams and Beer 2010; Rosas et al. 2019; Brenner et al. 2000).

In the same vein, regular graphs (using lines or arrows) are mostly used to represent pairwise relations (i.e., the isolated synaptic effect of each of the two LGN neurons onto the V1 neuron they converge on). A hyperedge (e.g., a triangle or a triangular arrow to indicate direction) can graphically represent the joint/synergistic *combined* effect of both presynaptic neurons acting in synchronous concert onto the postsynaptic V1 neuron (Figure 3b). Note that the pairwise graph is not entirely inadequate in that it still accounts for ∼60% of the joint effect. This partial adequacy might explain the persistence of strictly pairwise representations in neuroscience. A completely inadequate notation would long have been noted and replaced. However, a notation that (incompletely) captures some or most of the effect is easier to retain, leaving higher order residuals invisible by convention.

Technically, one can use a regular (pairwise) graph to represent the same hyperedge (Peixoto et al. 2026). This can be achieved via a bipartite graph that lists all nodes (neurons in our case) as a left‐hand column and all of their possible interactions, including the more‐than‐pairwise interactions as another set of nodes on the right‐hand side. The hyperedge described above then can be represented via edges that link all three of these neurons to a node that specifies their three‐way interaction. However, this bipartite representation requires interaction nodes that have no anatomical referent (i.e., an added node within the model that is not in itself a neuron, but a symbolic representation of their interaction). Hyperedges over the actual neurons do not suffer this problem. A hyperedge can depict ternary (and possibly also quaternary) interactions within the same format as standard diagrams of neuronal circuit anatomy.

In other words, once we examine causal (*effective*) *connectivity* between neurons (or any other neuronal structure that could give rise to more‐than‐pairwise interactions), augmenting standard graphs with hypergraphs can help capture such irreducible non‐pairwise, higher arity relations described by Alonso and coworkers.

Early work on neuronal population dynamics softened concerns regarding the application of a strictly pairwise approach to neuronal data in that adding higher order (higher arity) interactions to computational models did little to improve their descriptive performance (Schneidman et al. 2006). However, several subsequent studies, including one from the same authors, found that this original finding was largely due to limited sample sizes. Several studies found that once neuronal populations are observed on a large enough scale, higher order (higher arity) interactions rise in importance (Ganmor et al. 2011; Roudi et al. 2009; Ohiorhenuan et al. 2010; but see Nozari et al. 2024; Rosch et al. 2024 for opposing evidence). In other words, there is growing evidence that higher order (higher arity) neuronal interactions occur in the central nervous system. At the same time, many currently applied techniques, such as strictly pairwise statistics and strictly pairwise representations of neuronal connectivity that do not account for these higher order effects could allow for a methodological (and/or a conceptual) blind spot for neuronal population processes (James et al. 2016; Stramaglia et al. 2014; Williams and Beer 2010; Rosas et al. 2019; Brenner et al. 2000).

To summarize, empirical studies have shown that simultaneous activation of two input neurons can produce an effect in a third downstream neuron that is not predictable from the effects of each input‐output pair in isolation (Alonso et al. 1996). In other words, the output is not a simple addition (or multiplication) of pairwise interactions but reflects *additional information* embedded in the timing of inputs. Studies that explicitly tested neuronal recordings of neuronal populations confirm that such higher order effects are of significance (Ganmor et al. 2011; Roudi et al. 2009; Ohiorhenuan et al. 2010).

## 4 Hypergraphs and Simplicial Complexes for Neural Connectivity

As stated above, higher order relations (interactions) of neurons can be represented by augmenting standard graph theory with hypergraphs that visually capture more‐than‐pairwise relations in a way that is suitable for neuroscience. Using hypergraphs (and related topological structures, such as simplicial complexes) might help expand systems neuroscience from uni‐ or bivariate time series analyses (Leung et al. 2025; Cliff et al. 2023) toward higher order neuronal data analyses. Simplicial complexes are a special class of hypergraphs in which inclusion of a higher order edge entails inclusion of all of its lower‐dimensional edges. Simplicial complexes thus are especially well suited to represent higher order neuronal interactions that coexist with their constituent pairwise interactions, such as Alonso et al.’s neuronal triangle discussed above.

There is increasing appreciation of adding *neuronal connectivity* to the study of neuronal function because *neuronal activity* alone is insufficient to understand brain function (Gallego 2025; Perich et al. 2025; Ebitz and Hayden 2021). It takes the context of neuronal connectivity to translate neuronal activation into causal (mechanistic) processes that underpin information transfer and processing. One way to think about neuronal connectivity thus is the potential for causal effects across individual neurons (Grasso et al. 2021). However, many traditional techniques of mapping neuronal connectivity are based on anatomical connectivity, which provides limited insight into causal efficacy between neurons (Schölvinck et al. 2010). By its very nature, anatomical neuronal connectivity is reducible to pairwise connections, and standard graph theory has proven well suited to represent the associated patterns (Bullmore and Sporns 2009; Seguin et al. 2023; Sporns 2018).

In more recent years, anatomical connectivity has been augmented by estimates of causal impact between (groups of) neurons based on the spread of neuronal activation. First among these techniques is “functional connectivity,” which translates consistent co‐activation (correlation) among neuronal structures with a notion of (underlying) connectivity (Snyder and Bauer 2019; Frostig et al. 1983; Biswal et al. 1995; Michalski et al. 1983). Another measure in this vein is “effective connectivity,” which goes beyond correlational observations (Friston 2011; Aertsen et al. 1989; Horwitz 2003; Reid et al. 2019). Specifically, effective connectivity can be defined as the evoked activation in (target) neuronal structures that consistently arises following artificial stimulation of a (source) neuronal structure (Massimini et al. 2005).

In the context of this study, it is worth noting that *both* functional and effective connectivity, as activation‐based measures, are affected by the limitations discussed above: Since the simultaneous effect of two or more (source) neuronal structures onto a converged‐upon target structure can be highly non‐linear, estimating this synergistic effect from isolated pairwise data can be insufficient. As a corollary, standard graph theory struggles to represent such synergistic effects of two or more presynaptic upstream neurons (or areas) on a postsynaptic downstream neuron (or area)—unless a bipartite representation is chosen which abstracts away from physical space.

We thus propose to augment standard graph theory with hypergraphs and simplicial complexes (Figure 4). Hypergraphs and simplicial complexes can augment a strictly pairwise graph‐theoretical approach by using diagrams that use surfaces and shapes in addition to lines to represent higher order interactions between functionally or effectively connected neuronal structures (Figure 5).

**FIGURE 4:**
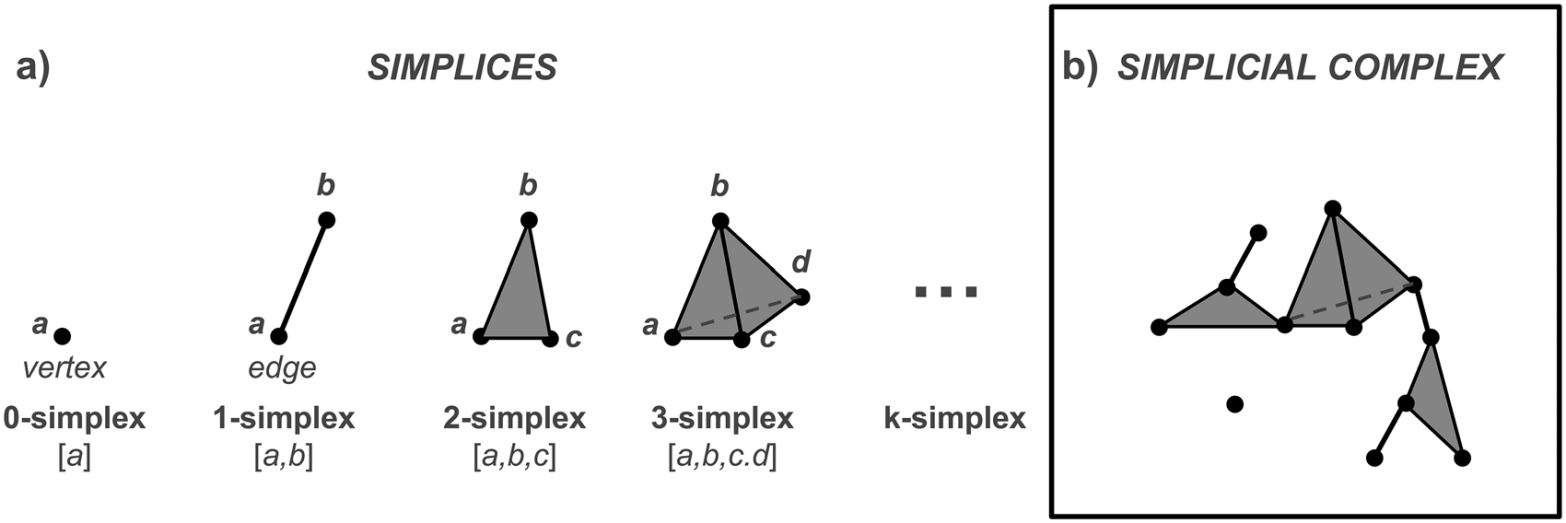
Simplicial complexes: a special type of hypergraph that lends itself to neuroscience. (a) Simplexes. A simplex is a generalization of triangles to arbitrary dimensions (i.e., a simplex represents the simplest possible polytope in any given dimension). A zero‐dimensional simplex is a point, a 1‐dimensional simplex is a line segment, a two‐dimensional simplex is a triangle, a three‐dimensional simplex is a tetrahedron, and so on. (b) Simplicial complexes. A geometric simplicial complex is a structured set of simplices such that all the faces and intersections of the elements are also included in the set.

**FIGURE 5:**
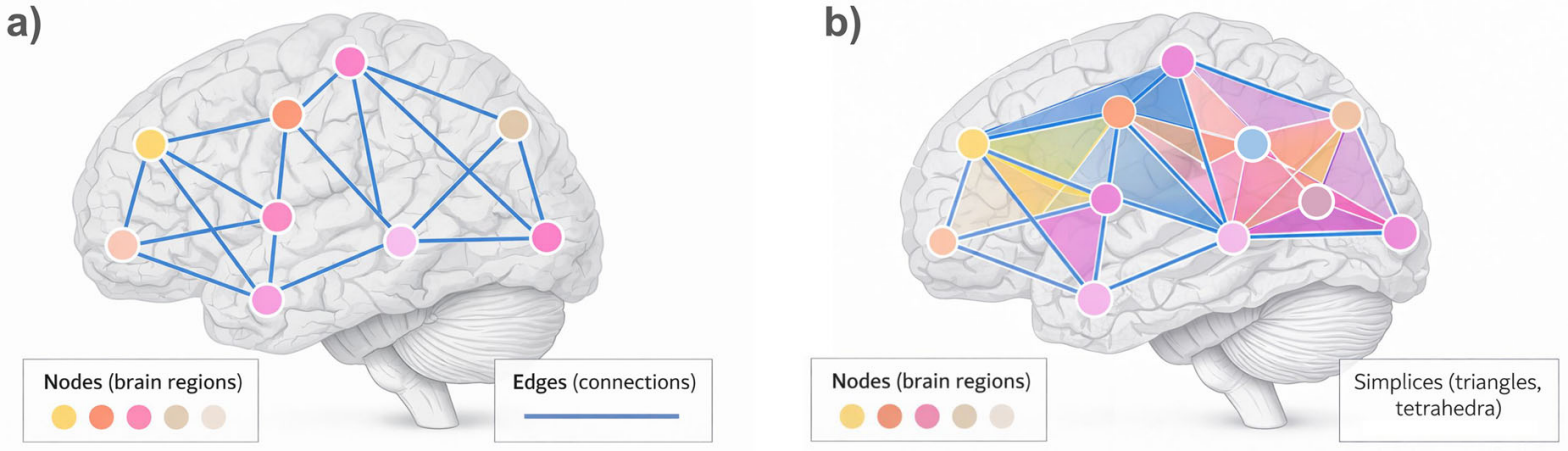
Extending graphs to simplicial complexes for neuronal connectivity. (a) A standard graph presentation of neuronal connectivity is limited to pairwise relations. (b) A simplicial complex representation of neuronal connectivity can more richly capture both pairwise and non‐pairwise (ternary and even quaternary) types of functional or effective connectivity.

One potential benefit of using hypergraphs to denote higher order (higher arity) neuronal interactions is conceptual. Notation can affect how we think about formal problems (Iverson 2011; Colyvan and Hall 2026; Colyvan 2020). Thus, expanding diagrams of neuronal connectivity from strictly pairwise notation to visual representations of (more‐than‐two‐neurons) collective causal action might be helpful to remind and steer neuroscientific thought into that direction. However, there also is an increasing body of work that aims to leverage hypergraph representations, and related expansions from two‐dimensional matrix‐based data analyses to higher dimensional hypermatrix or tensor‐based data analysis, more directly (Shetty and Arathi Bhat 2025; Bianconi and Dorogovtsev 2024; Zlatić et al. 2009; Saracco et al. 2025; Preti et al. 2024; Chodrow et al. 2021; Chodrow 2020; Young et al. 2017; Rosas et al. 2019; Santoro et al. 2023; Delabays et al. 2025).

Hypergraphs and simplicial complexes are a developing research area, but there already is an increasing number of computational tools available (Pickard et al. 2023; Marinazzo et al. 2024; Schaub et al. 2021; Wasserman 2018). In fact, neuroscientific studies are already making use of these mathematical tools (in the form of so‐called topological data analysis, TDA) in contexts other than studies of connectivity (Rouse et al. 2023; Wasserman 2018).

## 5 Empirical Demonstrations of Applicability and Benefits

As stated above, the adoption of hypergraph‐based approaches (or related attempts at capturing and studying more‐than‐pairwise neuronal interactions) is only starting to gain traction (e.g., Kurkin et al. 2026; Yu et al. 2011; Petri et al. 2014; Expert and Petri 2022; Camino‐Pontes et al. 2025; Santoro et al. 2025; Watson et al. 2026). There nonetheless already are several noteworthy demonstrations of both applicability and potential benefits worth highlighting. This is important since any hypergraph‐based approach to real‐world data needs to be compared to graph‐based alternatives to establish validity (Peixoto et al. 2026).

For example, Delabays et al. (2025) recently developed a novel model‐free technique that derives hypergraphs and simplicial complexes from (neural) time series data. They first established validity by testing their algorithm against synthetic data before applying it to resting state electroencephalographic (EEG) data to derive functional connectivity. Their main finding was that 60%–70% of the inferred macroscopic brain dynamics could be attributed to more‐than‐pairwise interactions. However, inference of functional connectivity from EEG data is inherently limited (e.g., Kajikawa and Schroeder 2011; Leopold and Maier 2012; Herrera et al. 2022). Indeed, the authors caution that higher order structures detected in EEG (or functional magnetic resonance imaging [fMRI]) do not allow for inferences about convergent anatomical circuits. Nonetheless, this finding suggests that higher order processes may be of significance to studying neuronal dynamics.

Santoro et al. (2024) used higher order approaches to analyze fMRI time series from the Human Connectome Project. They found that taking more‐than‐pairwise interactions into account enhanced both decoding of tasks as well as behavior (i.e., variations in cognitive performance) from brain activity compared to conventional pairwise statistical techniques.

Of note, most existing studies that apply hypergraph‐based, or related higher order, analyses to neuronal data are limited to indirect measures of neuronal communication using EEG, fMRI, or similar population signals (Kurkin et al. 2026). A major step to move from the detection of higher order *correlations* to the identification of higher order *causal* mechanisms would be to study neuronal triplets with identified monosynaptic connections, ideally augmented by causal interventions such as optogenetic activation. This seems methodologically in reach for smaller nervous systems, such as that of *Caenorhabditis elegans* (Randi et al. 2023). In mammals, it is already feasible to detect putatively monosynaptically connected neuronal pairs within the hundreds of simultaneously measured single neuron responses that set the current state of the art (Ren et al. 2022). As technological advances increase the number of simultaneously measured neurons, the likelihood of detecting monosynaptically connected neuronal triplets should increase as well.

## 6 Discussion

As demonstrated by the example of Borromean rings, some physical phenomena do not exist in any pairwise subset. A ternary neural event marks a similar physical occurrence that may not be fully reducible to a pairwise sequence. A measurable consequence of the type of higher order processes we discussed then is that strictly pairwise statistics (standard correlations, conventional Granger causality) will systematically miss irreducible non‐pairwise effects that are demonstrable in neural tissue (James et al. 2016; Stramaglia et al. 2014; Williams and Beer 2010; Rosas et al. 2019; Brenner et al. 2000). Hypergraphs and simplicial complexes provide a convenient visual aid to represent such higher order relations.

The advent of linear multielectrode arrays yielding hundreds or even thousands of neuronal activity measurements at a time has provoked a shift in data analysis to more structural descriptions, such as geometric manifolds and topological data analysis (Ebitz and Hayden 2021; Gallego 2025; Perich et al. 2025; Giusti et al. 2016; Mitchell‐Heggs et al. 2023; Gardner et al. 2022; Ruff et al. 2018; Rouse et al. 2023). At the same time, structural approaches have also enjoyed renewed popularity in related fields such as psychophysics, which some authors have coined a “structural turn” (Maier and Tsuchiya 2026; Kleiner 2024; Lee 2021).

Some of these structural approaches, resting on higher arity relations, have started to exploit the tight relationship between brain structure and brain function to *predict* neuroanatomical structure based on the outcome of neuronal processing, such as during sensation and perception (Grasso, Haun, et al. 2021; Comolatti et al. 2025). Approaches beyond pairwise neuronal action are arguably growing in relevance as we are leaving behind the technical limitation of measuring one or few neurons at a time.

We are rapidly entering a new age in systems neuroscience. We not only analyze the activity of thousands of neurons at a time, but we increasingly do so via free, open, and team‐based research in order to manage the associated workload (Aizenbud et al. 2025). New paradigms require new approaches. Using this opportunity to follow Jon Kaas’ vision of an integrated approach that examines brain function within a structural context to understand how “structure guides function” seems promising.

## Conflicts of Interest

The author declares no conflicts of interest.
